# Development of a cost‐effective single nucleotide polymorphism genotyping array for management of greater yam germplasm collections

**DOI:** 10.1002/ece3.5141

**Published:** 2019-04-29

**Authors:** Fabien Cormier, Pierre Mournet, Sandrine Causse, Gemma Arnau, Erick Maledon, Rose‐Marie Gomez, Claudie Pavis, Hâna Chair

**Affiliations:** ^1^ CIRAD, UMR AGAP Petit‐Bourg France; ^2^ CIRAD, INRA, Univ Montpellier, Montpellier SupAgro Montpellier France; ^3^ CIRAD, UMR AGAP Montpellier France; ^4^ INRA, UR ASTRO Agrosytèmes Tropicaux Petit‐Bourg France

**Keywords:** *Dioscorea alata* L., ex situ collection, genotyping, KASPar, ploidy, yam

## Abstract

Using genome‐wide single nucleotide polymorphism (SNP) discovery in greater yam (*Discorea alata* L.), 4,593 good quality SNPs were identified in 40 accessions. One hundred ninety six of these SNPs were selected to represent the overall dataset and used to design a competitive allele specific PCR array (KASPar). This array was validated on 141 accessions from the Tropical Plants Biological Resources Centre (CRB‐PT) and CIRAD collections that encompass worldwide *D. alata* diversity. Overall, 129 SNPs were successfully converted as cost‐effective genotyping tools. The results showed that the ploidy levels of accessions could be accurately estimated using this array. The rate of redundant accessions within the collections was high in agreement with the low genetic diversity of *D. alata* and its diversification by somatic clone selection. The overall diversity resulting from these 129 polymorphic SNPs was consistent with the findings of previously published studies. This KASPar array will be useful in collection management, ploidy level inference, while complementing accurate agro‐morphological descriptions.

## 1 INTRODUCTION

Greater yam (*Discorea alata* L.) is one of the major cultivated yam species (*Discorea *spp.) and the most widely spread among tropical and subtropical regions. The high importance of *D. alata* for food security has prompted the establishment of several international and national ex situ collections. Due to the limited shelf‐life of stored tuber, yam genetic resources are conserved in vitro or/and in the field. All of these repeated manipulations are time‐consuming and may affect long‐term conservation. Quality control of genotype purity and general collection management is mainly based on morphological descriptors (IPGRI/IITA, 1997; Mahalakshmi et al., 2007). However, these descriptors are not reliable enough to rationalize ex situ* D. alata* collection. Indeed, several studies have revealed that morphological variations are not necessarily linked to geographic origin or genetic lineage (Arnau et al., 2017; Lebot, Trilles, Noyer, & Modesto, 1998; Vandenbroucke et al., 2016). Complementary characterization tools are thus required for the conservation and dynamic management of ex situ collections related to germplasm exchange, the development of core collection or identification of future parents for breeding programs. *D. alata* is also a polyploid species with ploidy levels of 2*n* = 2*x*, 3*x*, or 4*x* and a basic chromosome number of *x* = 20 (Arnau, Némorin, Maledon, & Abraham, 2009). Ploidy levels detection is consequently a prerequisite for the identification of possible parents as crosses between the different ploidy levels can fail (Nemorin et al., 2013).

Molecular markers have been used to characterize *D. alata* diversity: random amplified polymorphic DNA (RAPD; Asemota, Ramser, Lopez‐Peralta, Weising, & Kahl, 1996), isoenzymes (Lebot et al., 1998), amplified fragment length polymorphism (AFLP; Malapa, Arnau, Noyer, & Lebot, 2005), simple sequence repeats (SSRs; Siqueira, Marconi, Bonatelli, Zucchi, & Veasey, 2011; Sartie, Asiedu, & Franco, 2012; Otoo, Anokye, Asare, & Telleh, 2015; Chaïr et al., 2016; Arnau et al., 2017), plastid sequences (Chaïr et al., 2016), and Diversity Arrays Technology (DArT; Vandenbroucke et al., 2016). These studies generated essential information on the diversity and representativity of the germplasm collections. However, these tools were not tailored for routine collection management. They were found to be either poorly discriminating within *D. alata* species or they were complex and not cost‐effective to use. Besides the development of high‐throughput methods for genome‐wide variant detection, such as genotyping‐by‐sequencing (Davey et al., 2011) paired with cost‐effective SNP assay (Broccanello et al., 2018) as KASPar can lead to the development of appropriate markers for collection management. This approach has been successfully implemented in maize (Semagn et al., 2012), chickpea (Hiremath et al., 2012), *Citrus* (Garcia‐Lor, Ancillo, Navarro, & Ollitrault, 2013), pigeon pea (Saxena et al., 2014), and *Brassica rapa* (Su et al., 2018). Regarding the recent release of yam (*Dioscorea *spp.) genomic resources (Saski, Bhattacharjee, Scheffler, & Asiedu, 2015; Tamiru et al., 2017), the design of such markers for *D. alata* collection management would be worthwhile. Indeed, once developed they do not require any specific bioinformatics or wet chemistry skills. The results contain few erroneous and missing data and can be easily analyzed and interpreted.

The main objectives of this study were (a) to identify genome‐wide polymorphic SNP markers, (b) to develop a cost‐effective SNP genotyping array using KASPar technology and (c) to test its use as a tool in managing yam ex situ collections.

## 2 MATERIALS AND METHODS

### 2.1 Materials

Based on a previous microsatellite markers study (Arnau et al., 2017), a set of 48 accessions representing worldwide *D. alata* diversity was selected and genotyped to identify polymorphic SNPs and design KASPar markers. Then, for the purpose of validating these markers, 141 landraces from the Tropical Plants Biological Resources Centre (CRB‐PT) and CIRAD ex situ collections maintained in the West French Indies (Guadeloupe) were used.

### 2.2 Genotyping‐by‐sequencing (GBS) and SNP discovery

SNP discovery was based on genotyping‐by‐sequencing (GBS). First, DNA extractions were performed with dried leaves from the 48 accessions as described by Risterucci et al. (2009). The genomic DNA quality was checked using agarose gel electrophoresis, and the quantity was estimated using a Nanodrop ND‐1000 spectrophotometer (Thermo Scientific, Wilmington, USA). For GBS, a genomic library was prepared using the PstI‐MseI restriction enzymes (New England Biolabs, Hitchin, UK) with a DNA normalized quantity of 200 ng per sample. The procedures published by Elshire et al. (2011) were adapted as described in Cormier et al. (2019).

Digestion and ligation reactions were conducted in the same plate. Digestion was conducted at 37°C for 2 hr and then 65°C for 20 min to inactivate the enzymes. The ligation reaction was achieved using T4 DNA ligase enzyme (New England Biolabs, Hitchin, UK) at 22°C for 1 hr, and the ligase was then inactivated, prior to sample pooling, by heating at 65°C for 20 min. Pooled samples were PCR‐amplified in a single tube. Single‐end sequencing was performed on a paired‐end lane of an Illumina HiSeq3000 (at the GeT‐PlaGe platform, Toulouse, France). The Tassel 5.2 pipeline (Glaubitz et al., 2014) was used for SNP and indel calling. Sequence tags were aligned to *D. alata* contigs (http://www.ebi.ac.uk/ena/data/view/PRJEB10904) using Bowtie2 v2.2.6 (Langmead & Salzberg, 2012). Accessions with more than 70% missing data were removed. Vcf filtering was performed using Vcftools 0.1.14 (Danecek et al., 2011; option: ‐‐minDP 8, ‐‐maf 0.1, ‐‐max‐missing 0.60, ‐‐max‐alleles 2, ‐‐thin64).

### 2.3 KASPar genotyping and allele calling

Polymorphic SNP flanking sequences (60 bp upstream and 60 bp downstream around the variant position) were selected using SNiPlay3 (Dereeper et al., 2011). In order to assess their putative physical positions, these sequences were then blasted to the *D. rotundata* reference genome (TDr96_F1 Pseudo_Chromosome: BDMI01000001–BDMI01000021; Tamiru et al., 2017). The physical position of each SNP was defined using their flanking sequences best hit using a BLAST E‐value threshold of 1e−30 (Basic Local Alignment Search Tool). Finally, 192 SNPs were selected by forming 192 k‐means cluster based on their relative physical distance (Euclidean distance) and selecting the SNP nearest to the centroid of each cluster using R 3.4.0 (R core team, 2017).

The 192 SNPs were converted into a KASPar assay at LGC genomics where the primer design and wet chemistry was conducted (Middlesex, UK) on a validation panel of 141 landraces from the CRB‐PT and CIRAD ex situ collections. From raw fluorescence data, allele calling was performed using LGC Kluster Caller software by defining fluorescence clusters. Some accessions with known ploidy level were used as reference to identify fluorescence clusters and assess allelic dosage.

### 2.4 Diversity analysis

To identify duplicate accessions and compare accessions with different ploidy levels, a matrix of dissimilarity between each accession pair was computed as the percentage of shared alleles based on the allele presence/absence.

Then, to refine the kinship assessment, similarities between accessions with the same ploidy level were computed in the same way but using the allelic dosage. For diploid accessions, genotypes were coded as 0, 1, and 2 where the number represents the number of nonreference allele. Heterozygous genotypes assessed as polyploid during allele calling were converted to 1. Moreover, for triploid accessions, genotypes were coded as 0, 1, 2, and 3 with allelic dosage score as 1:1 during allele call converted to 1.5. For tetraploid accessions, genotypes were thus coded as 0, 1, 2, 3, or 4 and no correction was needed.

Diversity analysis was conducted in two steps. During the first step, groups of duplicate accessions (redundancy groups) were defined by grouping accessions having up to one allele mismatch. Then, in the second step, the diversity analysis focused on the similarity between those groups. Clustering based on allele frequencies within redundancy groups followed by a bootstrap approach (pvclust R package, ward.D2, 10,000 boots, AU threshold = 0.95; Suzuki & Shimodaira, 2006) was used to identify gene pools. A diversity network between redundancy groups was also drawn using significant kinship detected through genotype permutations (1,000), with a significance threshold of 0.05.

## 3 RESULTS

### 3.1 KASPar assay development and validation

Genotyping‐by‐sequencing (GBS) produced more than 344 million reads resulting in 521,918 sequence tags out of which 207,810 (39.82%) aligned exactly once on *D. alata* contigs. The remaining reads aligned at multiple locations (25.18%) or did not align to any contig (35%). From these sequence tags, SNP calling produced a raw vcf file of 158,695 SNPs. This raw vcf file was then filtered resulting in a dataset of 40 accessions (Appendix A), and 4,593 good quality SNPs out of which 3,879 (84%) SNPs were mapped by BLAST on the *D. rotundata* reference genome. The KASPar assay was then developed by selecting 192 SNPs representative of SNPs mapped along the *D. rotundata* reference sequence, and they were tested on 141 accessions.

Among the 192 SNPs, 26 (13%) SNPs failed as they did not produce any amplification signal. From the remaining 166 SNPs (87%), 129 SNPs (Appendix C) with less than 20% missing data and a minor allele frequency of over 5% were retained as high‐quality SNPs. This final dataset (129 SNPs × 141 accessions) contained an overall missing data rate of only 0.5% with a maximum of 3% missing data per accession.

**Figure 1:**
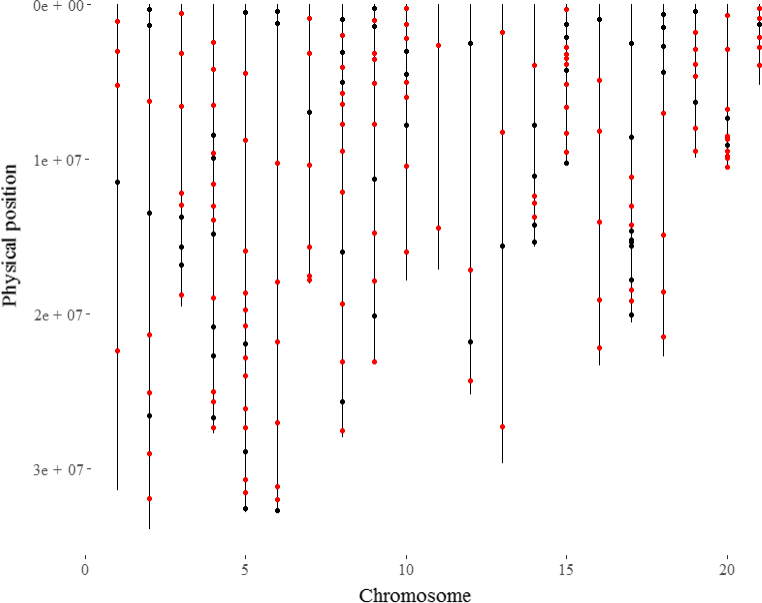
Location of KASPar SNPs on the *D. rotundata* reference genome (Tamiru et al., 2017). The 21 linkage group are aligned from left to right. Black dots, failed or bad quality SNPs; red dots, the 129 validated SNPs

The 129 validated KASPar SNPs were distributed on all linkage groups used to construct the *D. rotundata* reference genome (Figure 1). Their distribution was not homogeneous along chromosomes as their position was planned to be representative of that of the initial set of 3,879 mapped SNPs and not equally spaced.

### 3.2 Assessment of ploidy levels

In our *D. alata* validation panel, three ploidy levels (2*x*, 3*x* and 4*x*) coexisted (Appendix B). Thus, the KASPar assay could theoretically produce a maximum of seven types of fluorescence signal (Table 1) corresponding to two types of fluorescence signal in homozygous states (2:0 = 3:0 = 4:0; 0:2 = 0:3 = 0:4), the fluorescence signal of mixed and balanced allelic dosages (1:1 for diploids or 2:2 for tetraploids) and the four types of fluorescence signal corresponding to the different possible unbalanced allelic dosages at heterozygotic loci (“polyploid‐like” in Table 1) of triploids and tetraploids (1:3; 1:2; 2:1; 3:1). In our case, due to insufficient fluorescence resolution, it was not possible to distinguish fluorescence signals of the 1:3 tetraploid allelic dosage from the 1:2 triploid allelic dosage, or the 2:1 triploid allelic dosage from the 3:1 tetraploid allelic dosage. Consequently, a maximum of five types of fluorescence signals were identified. Overall, five, four, three, and two allelic dosages were detected for 64 (50%), 41 (32%), 19 (15%), and 5 (4%) SNPs, respectively, because some allelic dosages were not present in the validation panel or they were cofounded.

**Table 1:** Summary of genotype, allelic composition and fluorescence signals

Type of genotype	Ploidy	Allelic		Type of fluorescence signal	
		Dosage	Composition	Theo.	Obs.
Diploid‐like	Diploid	0:2	X:X	1	1
		1:1	X:Y	4	3
		2:0	Y:Y	7	5
	Triploid	0:3	X:X:X	1	1
		3:0	Y:Y:Y	7	5
	Tetraploid	0:4	X:X:X:X	1	1
		2:2	X:X:Y:Y	4	3
		4:0	Y:Y:Y:Y	7	5
Polyploid‐like	Triploid	1:2	X:X:Y	3	2
		2:1	X:Y:Y	5	4
	Tetraploid	1:3	X:X:X:Y	2	2
		3:1	X:Y:Y:Y	6	4

**Figure 2:**
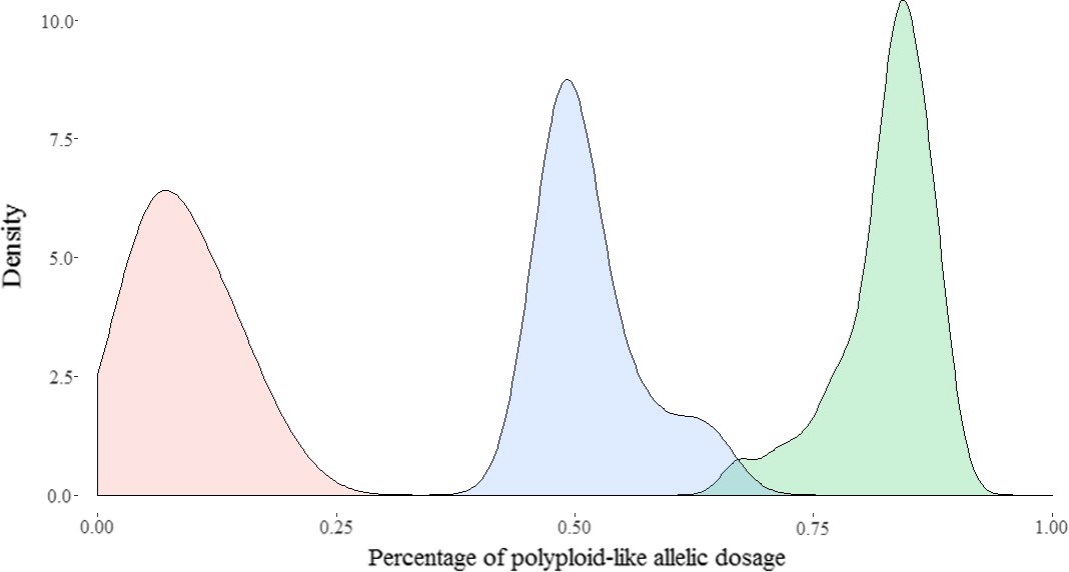
Distribution of the percentage of polypoid‐like genotypes (1:3, 1:2, 2:1, and 3:1 allelic dosage) on overall heterozygous genotypes by ploidy level (red, diploid; green, triploid; blue, tetraploid)

However, the overall allele call and allelic dosage assessment quality were good. Indeed, the ratio of genotypes scored as “polyploid‐like” on overall heterozygous genotypes by accession was low (0.09 ± 0.05) for diploids and high for triploids (0.83 ± 0.05). In addition, the three distributions of this ratio corresponding to the three ploidy levels did almost not overlap (Figure 2).

We were thus not able to differentiate all allelic dosage from each other when looking at one SNP. However, ploidy level could be deduced when taking all the KASPar array into account and considering the proportion of genotypes scored as “polyploid‐like” per accession. This KASPar assay thus differentiated the accession ploidy level and allowed us to assign it for 12 accessions originally of unknown ploidy. Nine were set as diploid and three as triploid.

### 3.3 Diversity analysis

Overall, 141 accessions from CRB‐PT and CIRAD ex situ collections in Guadeloupe were used to validate the KASPar assay (96 diploids, 36 triploids, and nine tetraploids including accessions with known and deduced ploidy level).

**Figure 3:**
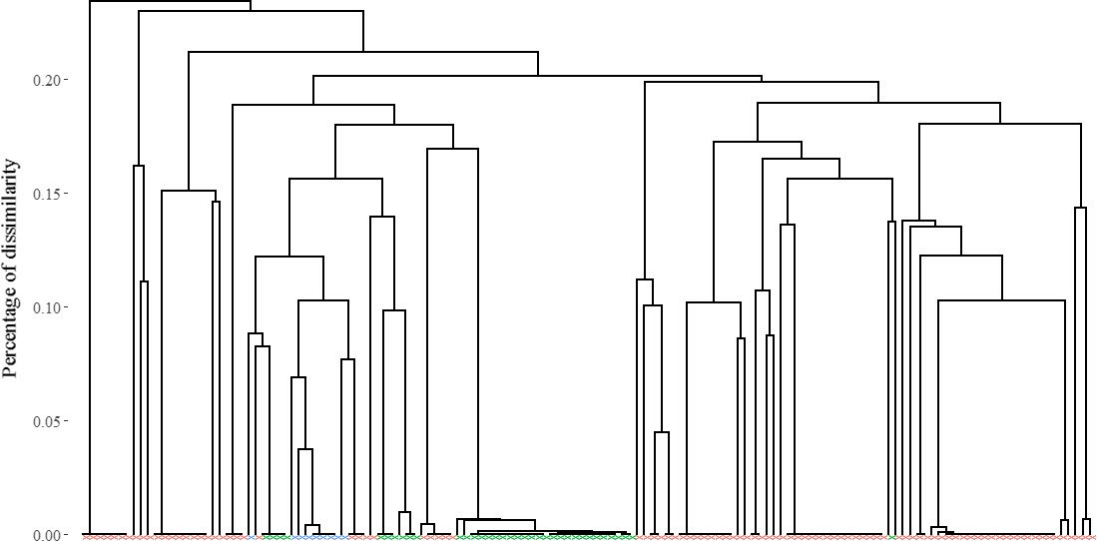
Dendrogram of dissimilarity between 141 *D. alata* accessions (red, diploid; green, triploid; blue, tetraploid)

The allele presence and/or absence was used to assess the similarity between accessions and thus to identify duplicate accessions (Figure 3). Indeed, by defining redundancy groups, we ended up with 43 nonredundant groups each containing one to 24 accessions.

These groups of genetically similar accessions were partially expected based on the accession vernacular names. For example, the second biggest group (redundancy group 6, Appendix B) was composed of 18 accessions, five of which had a name related to “Saint Vincent.” The third biggest group contained 14 accessions, four of which had a name related to “Pacala.”.

The main group of redundant accessions was composed of 24 triploids collected at several distant locations (Caribbean islands, New Caledonia and Madagascar). This group consisted of 67% (24/36) of the triploid accessions present in the CRB‐PT and CIRAD collections.

**Figure 4:**
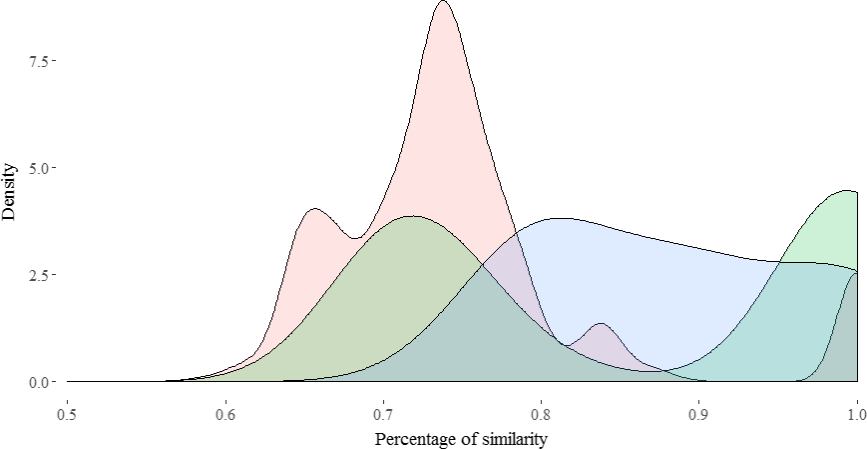
Distribution of similarity between all accession pairs by ploidy (red, diploid; green, triploid; blue, tetraploid)

More generally, redundancy groups only consisted of accessions with the same ploidy level (Figure 4). Moreover, similarities within triploids or within tetraploids were higher than within diploids.

**Figure 5:**
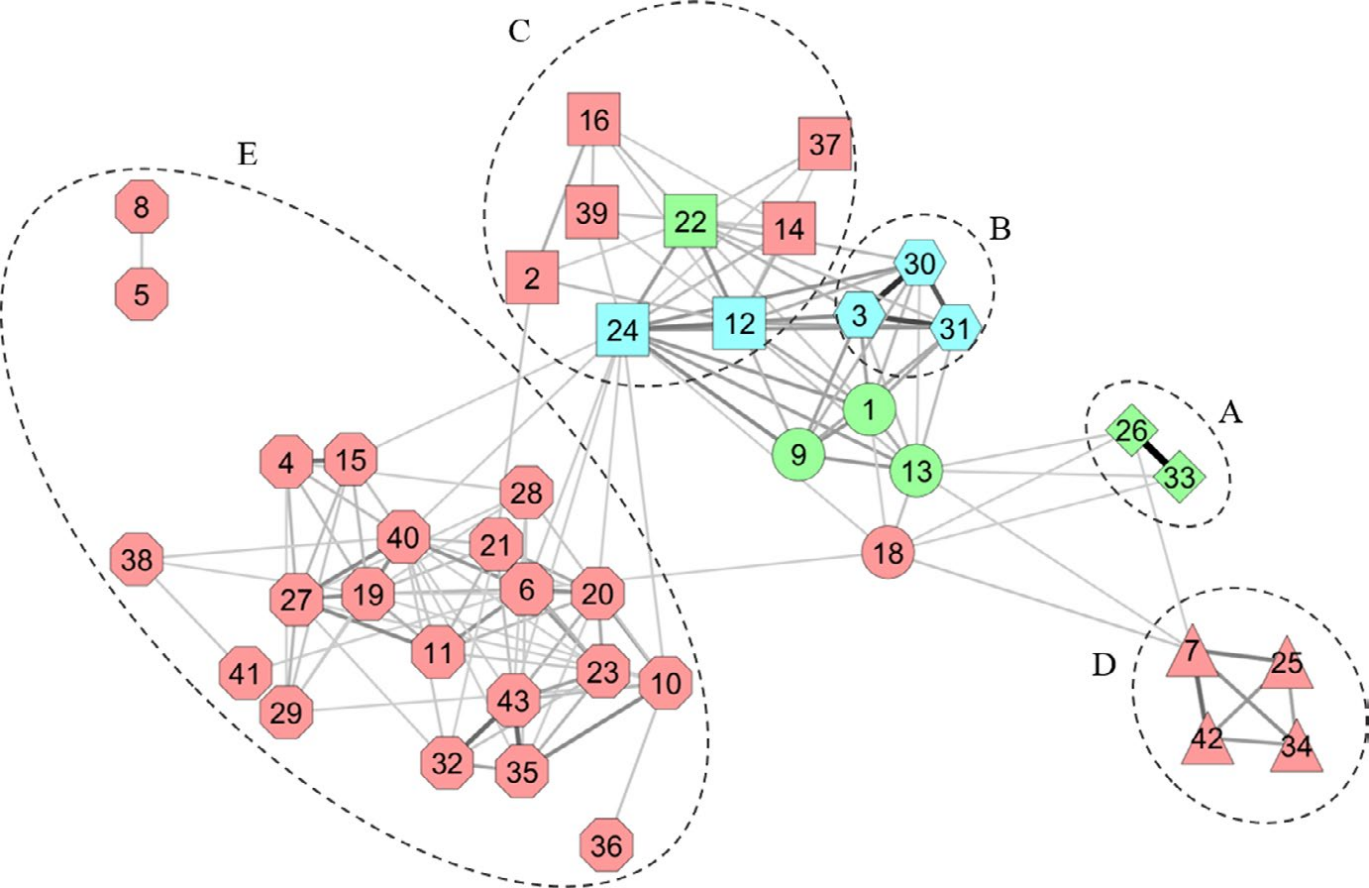
Network of kinship for the 43 *D. alata* redundancy groups based on significant similarity (*p* < 0.05, edge‐weighted spring‐embedded layout). Nodes shape and letter, cluster of diversity identified by a bootstrap procedure; red nodes, diploids; green nodes, triploids; blue nodes, tetraploids; edge colors, similarity from gray (0.64) to black (1)

The diversity analysis was based on these 43 redundancy groups to avoid bias. After clustering, the bootstrap procedure detected five significant gene pools, named “cluster” here, represented in the kinship network (Figure 5). Only one (cluster C, Figure 5) consisted of accessions from the three ploidy levels. This cluster encompassed accessions from the Caribbean and Pacific regions. Clusters A, B, and D contained triploids from the Caribbean and Madagascar, tetraploids from the Pacific and diploids from the Caribbean, respectively (Figure 5, Appendix B). Cluster E was the biggest one, with 21 nonredundant diploid accessions originating from India, Nigeria, Côte d'Ivoire, the Caribbean and Pacific (Figure 5, Appendix B).

Genotype permutations and network analysis gave a more detailed view of kinship between redundancy groups and Clusters. This approach revealed a low number of significant links between the diversity clusters D or E and the others (Figure 5) revealing that these clusters could consist of original genepools.

## 4 DISCUSSION

### 4.1 Assessment of allelic dosage and detection of ploidy levels

KASPar technology is based on competitive allele‐specific amplification followed by allele‐specific fluorescence assessment (Semagn, Babu, Hearne, & Olsen, 2014). Detection of allelic dosage in polyploid species is thus possible (Cuenca, Aleza, Navarro, & Ollitrault, 2013). However, several parameters may influence the fluorescence, such as the DNA quality or primer specificity, and consequently the ability to discriminate fluorescence signals and the allelic dosage. In our case, we were able to discriminate five types of fluorescence signal. At heterozygous loci, fluorescence signals were a mixture of two types of allelic‐specific fluorescence. Fluorescence signals should also be balanced for diploids which have a balanced allelic dosage (1:1) at heterozygous loci. Diploids should therefore theoretically have no genotypes assessed as “polyploid‐like.” Conversely, triploids should theoretically have only genotypes assessed as “polyploid‐like” at heterozygous loci. A balanced allelic dosage is impossible for triploids. Our results showed that 91 ± 5% and 83 ± 5% of heterozygous genotypes were correctly called for diploids and triploids, respectively. Regarding the recent explosion of genotyping related to next‐generation sequencing, bioinformatics tools have been developed to accurately determine dosages (e.g., GBS2ploidy; Gompert & Mock, 2017). However, this requires deep sequencing and usually an assumption of ploidy levels present in the dataset (Bourke, Voorrips, Visser, & Maliepaard, 2018).

Application in collection management may nevertheless not require allelic dosage assessment at each locus. Our aim was thus to develop a tool for estimating ploidy levels and not variations in copy number. Moreover, the results showed that ploidy levels for each accession can be accurately deduced from the percentage of “polypoid‐like” genotypes on overall heterozygous genotypes. Regarding the overlapping distributions of this ratio (Figure 2), the only risk is to confuse triploids and tetraploids estimated at 3%. Consequently, ploidy level assessment is possible and fairly accurate for *D. alata* using the KASPar assay developed in this study.

### 4.2 Identification of duplicate accessions

The dataset included 129 SNPs validated on 141 accessions corresponding to 43 unique redundancy groups. The resuming of the 141 accessions to 43 unique redundancy groups was related to the narrow *D. alata* genetic diversity, above all in polyploid germplasm (i.e., triploids and tetraploids) already identified in previous studies. For example, using DarT markers, a low varietal richness was revealed by Vandenbroucke et al. (2016), who studied 80 landraces from six different Vanuatu islands and differentiated only seven unique genotypes. Using isozyme markers, Lebot et al. (1998) studied 269 worldwide distributed cultivars and concluded that the genetic diversity of the most widespread cultivars was narrow.

Regarding the accession vernacular names, redundant accessions were expected in our sample. Some of these redundancy groups contained accessions detected in duplicate, while they could be differentiated by morphological characterization. For example, redundancy group five (including Lupias, Malalagi, or Malankon) exhibited diversity in tuber shape and tuber flesh color in agreement with previous genetic diversity studies that already pooled these accessions together and highlighted this intragroup variability in tubers (Arnau et al., 2017; Malapa et al., 2005).

Morphological variability within a redundancy cluster mostly arises via* D. alata* clonal reproduction and farmers' selection of new morphotypes resulting from somatic mutations (Lebot et al., 1998; Malapa et al., 2005; Vandenbroucke et al., 2016). Small genetic or epigenetic variations are commonly selected to create new diversity in horticultural crops such as yam as reviewed by Krishna et al. (2016).

The ability of KASPar assay developed in this study to differentiate duplicates in collections from genetically close accessions was related: (a) to the low number of studied loci (129), but also (b) to the *D. alata* diversification process (i.e., selection of somaclonal mutants) and (c) the presence of real duplicates within collections. This tool is thus efficient for attributing accessions to a genetic lineage (e.g., germplasm exchange), but a good complementary agro‐morphological and ecophysiological characterization of collections should also be done to completely differentiate somaclonal mutant clones from duplicates (e.g., identification of promising genitors for breeding programs).

### 4.3 Diversity and collection management

The CRB‐PT collection has been shown to be representative of worldwide *D. alata* diversity (Arnau et al., 2017). A subset of this ex situ collection has been genotyped in this study. However, all diversity groups identified by Arnau et al. (2017) were present (except one containing five very similar Indian accessions). Our validation panel was thus representative of the worldwide *D. alata* diversity. Moreover, a good correlation was obtained between the findings of the previous study of worldwide *D. alata* diversity of Arnau et al. (2017) and the gene pools identified in this study (Appendix B). We can thus hypothesize that the 129 SNPs KASPar array developed for *D. alata* allow us to accurately assess genetic diversity and the findings may be transferable to other collections. Moreover, this genotyping tool is a robust method: (a) to assess complementarity/redundancy between the different collections, (b) to identify under represented genetic groups, and (c) to plan future collects to fill gaps in collections.

## 5 CONCLUSION

This is the first SNP array designed for *D. alata* and validated on a subset of accessions representative of worldwide *D. alata* diversity. This tool will allow users to estimate accession ploidy levels and genetic lineages. The results showed a good correlation between the diversity assessed by this KASPar array and the findings of previous studies. This KASPar array is a robust and cost‐effective tool for diversity assessment and collections management. Regarding the importance of vegetative reproduction and somaclonal selection in *D. alata*, it is a good tool to complement agro‐morphological description in collections.

## CONFLICT OF INTEREST

The authors declare that they have no conflict of interest.

## AUTHOR CONTRIBUTIONS

C.P., F.C., H.C., and P.M. designed the study. C.P., F.C., E.M., G.A., and R‐M.G. contributed to collecting materials and sample preparation. P.M. and S.C. developed GBS protocol, carried out DNA extraction, and GBS library preparation. H.C. and P.M. performed SNP discovery. F.C. and H.C. designed the KASPar assay and performed its analysis. C.P., F.C., and H.C. wrote the manuscript with the input of all authors.

## Data Availability

Plant materials may be requested at the CRB‐PT of Guadeloupe http://intertrop.antilles.inra.fr/Portail/accessions/find/11. KASPar primers sequence is available in Appendix B.

## APPENDIX A

**Table A1:** Description of the 40 *D. alata* accessions used to detect polymorphic SNP

Collection	Code	Name	Origin	Ploidy
CRB‐PT	PT‐IG‐00002	Pakutrany	Nlle Caledonie	
	PT‐IG‐00006	Fénakué	Puerto Rico	2
	PT‐IG‐00010	Divin 1	Guadeloupe	2
	PT‐IG‐00020	DA 26	Guyane Fr	3
	PT‐IG‐00338	HYB 30	Guadeloupe	
	PT‐IG‐00350	Pacala	Guadeloupe	2
	PT‐IG‐00029	Plimbite	Haïti	2
	PT‐IG‐00033	Pyramide	Puerto Rico	2
	PT‐IG‐00046	Sea 190	Puerto Rico	2
	PT‐IG‐00053	Kokoéta	Nlle Calédonie	2
	PT‐IG‐00686	Roujol		4
	PT‐IG‐00687	INRA C 143		
	PT‐IG‐00688	INRA AL 56		
	PT‐IG‐00690	INRA AL 18		
	PT‐IG‐00692	INRA X 154	Guadeloupe	
	PT‐IG‐00693	INRA X 17	Guadeloupe	
	PT‐IG‐00694	Dou		4
	PT‐IG‐00695	INRA X 142	Guadeloupe	
	PT‐IG‐00696	Ciradienne		4
	PT‐IG‐00697	TiViolet		4
	PT‐IG‐00698	Malalagi	Vanuatu	2
	PT‐IG‐00702	Manlankon	Vanuatu	2
	PT‐IG‐00689	Nureangdan	Vanuatu	3
	PT‐IG‐00077	Kinabayo	Puerto Rico	2
	PT‐IG‐00078	Toro	Haïti	3
Cirad	Vu 024a	Tépuva	Vanuatu	2
	Vu 528a	Tacharamivar		2
	Vu 564a	Mendrovar	Vanuatu	2
	Vu 567a	Homb	Vanuatu	2
	Vu 754a	Intejegan	Vanuatu	4
	Vu 231a	Tagabé	Vanuatu	4
	Ovy taty		Madagascar	
	Vu 247a	*n*.a	Vanuatu	2
	Vu 401a	Basa	Vanuatu	2
	Kabusa			2
	74F			2
	42F			2
	61F			2
	14M			2
	H4x200			4

## APPENDIX B

**Table B1:** Description of the 141 *D. alata* used as the KASPar assay validation panel

Collection Code	Ploidy^a^	Div. Clust.^b^	Redund. Grp^c^	Accession name	Origin	SSR^d^
PT‐IG‐00087	3	A	26	65	Martinique	XII
PT‐IG‐00070	3	A	26	66	Martinique	XII
PT‐IG‐00090	3	A	26	Caillade 1	Haïti	XII
PT‐IG‐00020	3	A	26	DA 26	French Guyana	XII
PT‐IG‐00037	3	A	26	DA 27	French Guyana	XII
PT‐IG‐00022	3	A	26	De agua	Puerto Rico	XII
PT‐IG‐00061	3	A	26	Igname d eau	Martinique	XII
PT‐IG‐00550	3	A	26	Montpellier		XII
PT‐IG‐00075	3	A	26	Renta Yam	Jamaica	XII
PT‐IG‐00072	3	A	26	Sassa 1	Martinique	XII
PT‐IG‐00063	3	A	26	Sassa 2	Martinique	
PT‐IG‐00088	3	A	26	St Martin	Martinique	XII
PT‐IG‐00034	3	A	26	Sweet yam	Jamaica	XII
PT‐IG‐00557	3	A	26	Tahiti couleuvre	Guadeloupe	XII
PT‐IG‐00068	3	A	26	Tahiti cultivé	Guadeloupe	XII
PT‐IG‐00069	3	A	26	Tahiti French	Guadeloupe	XII
PT‐IG‐00018	3	A	26	Tahiti messien	Guadeloupe	
PT‐IG‐00064	3	A	26	Tana	New Caledonia	XII
PT‐IG‐00021	3	A	26	Telemaque	Martinique	XII
PT‐IG‐00044	3	A	26	Ti Joseph 1	Haïti	XII
PT‐IG‐00078	3	A	26	Toro	Haïti	XII
CT257_CIV	3	A	26	OvyTaty AmbalaKindresy‐Ambohimasoa	Madagascar	
CT258_CIV	3	A	26	OvyTaty Amboasary‐Ambohimasoa	Madagascar	
PT‐IG‐00685	*3*	A	26	Sainte Anne		
PT‐IG‐00030	3	A	33	67	Martinique	XII
PT‐IG‐00558	4	B	3	Wabé	New Caledonia	XVIII
Vu472a	4	B	3	Toufi Tetea	Vanuatu	XVIII
Vu231a	4	B	3		Vanuatu	XVIII
Vu750a	4	B	3	Wanorak	Vanuatu	
Vu534a	4	B	3	Bisoro	Vanuatu	XVIII
Vu754a	4	B	30	Noulelcae	Vanuatu	XVI
Vu408a	4	B	31	Manioc	Vanuatu	
PT‐IG‐00039	2	C	2	Americano	Dominican Republic	VII
PT‐IG‐00023	2	C	2	Florido	Puerto Rico	
PT‐IG‐00553	2	C	2	Pro 1		VII
PT‐IG‐00095	2	C	2	SEA 144	Puerto Rico	IV
PT‐IG‐00555	2	C	2	SRT 29		VII
PT‐IG‐00041	2	C	2	St Domingue	Dominican Republic	VII
Vu401a	2	C	2	Basa	Vanuatu	VII
CT256	*2*	C	2			
PT‐IG‐00009	4	C	12	Nouméa	New Caledonia	XVI
Vu247a	2	C	14		Vanuatu	
Vu528a	2	C	16	Sinoua	Vanuatu	
PT‐IG‐00025	3	C	22	Goana	New Caledonia	XIII
PT‐IG‐00002	3	C	22	Pakutrany	New Caledonia	XIII
Vu699a	*3*	C	22	Tumas	Vanuatu	
Vu461a	3	C	22	Tumas	Vanuatu	XIII
Vu755a	4	C	24	Nepelev	Vanuatu	
PT‐IG‐00014	2	C	37	Divin 2	Guadeloupe	
PT‐IG‐00006	2	C	37	Fénakué	Puerto Rico	
PT‐IG‐00053	2	C	37	Kokoéta	New Caledonia	
PT‐IG‐00559	2	C	39	Wassa	New Caledonia	
PT‐IG‐00001	2	D	7	64	Martinique	
PT‐IG‐00010	2	D	7	Divin 1	Guadeloupe	
PT‐IG‐00568	2	D	25	77	Martinique	IV
PT‐IG‐00092	2	D	34	Caplaou	Puerto Rico	
PT‐IG‐00561	2	D	42	H 23		
PT‐IG‐00562	2	D	42	H 50		
74F	2	E	4		India	
PT‐IG‐00049	2	E	5	Cinq	Puerto Rico	III
PT‐IG‐00027	2	E	5	Lupias	New Caledonia	III
PT‐IG‐00046	2	E	5	Sea 190	Puerto Rico	III
Vu590a	*2*	E	5		Vanuatu	III
Vu423a	2	E	5	Manlankon	Vanuatu	III
Vu639a	2	E	5	Malalagi	Vanuatu	III
Vu024a	2	E	5	Ptris	Vanuatu	III
PT‐IG‐00065	2	E	6	DA 28	French Guyana	IV
PT‐IG‐00093	*2*	E	6	DA 32		
PT‐IG‐00395	2	E	6	Fafadro bis		IV
PT‐IG‐00060	2	E	6	Grand Etang	Guadeloupe	IV
PT‐IG‐00051	2	E	6	Morado	Cuba	IV
PT‐IG‐00073	2	E	6	Purple Lisbon	Puerto Rico	IV
PT‐IG‐00333	2	E	6	Sainte Catherine	Guadeloupe	IV
PT‐IG‐00052	2	E	6	Smooth Statia	Puerto Rico	IV
PT‐IG‐00024	2	E	6	St Vincent blanc 1	Martinique	IV
PT‐IG‐00036	2	E	6	St Vincent blanc 2	Martinique	IV
PT‐IG‐00556	2	E	6	St Vincent mart.	Guadeloupe	IV
PT‐IG‐00045	2	E	6	St Vincent Violet	Martinique	IV
PT‐IG‐00016	2	E	6	St Vincent Yam	St. Lucia	IV
PT‐IG‐00374	2	E	6	Ti Joseph	Haïti	IV
PT‐IG‐00067	2	E	6	Wénéféla bis	New Caledonia	IV
Vu487a	2	E	6	Teroosi	Vanuatu	VI
770	*2*	E	6			
PT‐IG‐00623	*2*	E	6			
PT‐IG‐00396	2	E	8	A 24		
PT‐IG‐00071	2	E	10	72	Martinique	VIII
PT‐IG‐00055	2	E	10	76	Martinique	VIII
PT‐IG‐00089	*2*	E	10	Asmhore		
PT‐IG‐00058	2	E	10	Bété Bété	Côte d'Ivoire	VIII
PT‐IG‐00091	2	E	10	Campêche 2		
PT‐IG‐00546	2	E	10	Jardin Haitien		VIII
PT‐IG‐00547	2	E	10	Kourou 1	French Guyana	VIII
PT‐IG‐00548	2	E	10	Kourou 2	French Guyana	VIII
PT‐IG‐00350	2	E	10	Pacala	Guadeloupe	VIII
PT‐IG‐00551	2	E	10	Pacala cacao	French Guyana	VIII
PT‐IG‐00552	2	E	10	Pacala Guyane	French Guyana	VIII
PT‐IG‐00017	2	E	10	Pacala station	Guadeloupe	VIII
PT‐IG‐00554	2	E	10	SRT 24		VIII
19	*2*	E	10			
PT‐IG‐00057	2	E	11	Vino Purple forme	Puerto Rico	
61F	2	E	15		India	
PT‐IG‐00019	2	E	19	Gordito	New Caledonia	IX
PT‐IG‐00047	2	E	20	Buet	New Caledonia	
PT‐IG‐00029	2	E	20	Plimbite	Haïti	
PT‐IG‐00048	2	E	21	Bacala 1	Haïti	
PT‐IG‐00413	2	E	21	St Vincent	St. Vincent	
Cuba6	*2*	E	23		Cuba	
PT‐IG‐00542	2	E	27	AL 10		I
PT‐IG‐00042	2	E	27	Brazzo Fuerte	Puerto Rico	I
PT‐IG‐00038	2	E	27	Brésil 1		I
PT‐IG‐00564	2	E	27	KL 10		I
PT‐IG‐00565	2	E	27	KL 21		
PT‐IG‐00566	2	E	27	KL 40		I
PT‐IG‐00054	2	E	27	MP1 16H56		I
PT‐IG‐00033	2	E	27	Pyramide	Puerto Rico	I
PT‐IG‐00074	2	E	28	Oriental	Barbados	II
14M	2	E	29		India	
PT‐IG‐00077	2	E	32	Kinabayo	Puerto Rico	II
PT‐IG‐00085	2	E	35	St Sauveur	Guadeloupe	
PT‐IG‐00560	2	E	35	Yam jamaïque		
PT‐IG‐00543	2	E	36	Cross lisbon		
PT‐IG‐00392	2	E	38	A 13		
PT‐IG‐00398	2	E	38	A 2		
PT‐IG‐00563	2	E	40	Sc.c 1.1		
PT‐IG‐00008	2	E	41	AIA 445	Nigeria	
PT‐IG‐00015	2	E	43	Igname rouge	Guadeloupe	X
Vu703a	*3*	*F*	1	Nawanurunkimanga	Vanuatu	
PT‐IG‐00544	3	*F*	9	Cuello largo	Puerto Rico	XV
PT‐IG‐00026	3	*F*	9	Féo	Puerto Rico	XV
Vu696a	3	*F*	9	Nowateknempian	Vanuatu	XV
PT‐IG‐00076	3	*F*	13	Bélep	New Caledonia	XIV
Vu735a	3	*F*	13	Noplon	Vanuatu	XIV
Vu760a	3	*F*	13	Nureangdan	Vanuatu	XIV
PT‐IG‐00397	*2*	*F*	17	SEA 119, Toki		
Vu613a	2	*F*	17	Peter	Vanuatu	VI
Vu589a	2	*F*	17	Makila	Vanuatu	XI
VU590a	2	*F*	18		Vanuatu	III
Vu554a	2	*F*	18	Nourembor	Vanuatu	VI
Vu567a	2	*F*	18	Letsletsbolos	Vanuatu	IV
Vu564a	2	*F*	18	Makila	Vanuatu	VI
Vu026a	2	*F*	18	Dammasis	Vanuatu	VI

a In italic, ploidy detected using the percentage of polyploid genotype type on overall heterozygous loci.

b Group of diversity from diversity analysis

c Group of similarity used to select nonredundant accessions. Genotypes in the same group have a maximum of one allele mismatch).

d Cluster of diversity identified by SSR in Arnau et al. (2017).

## APPENDIX C

**Table C1:** KASPar assay description for the 129 high‐quality SNPs: Number of fluorescence type detected, chromosome and position on *D. rotundata* reference genome assessed by BLAST (*E*‐value)

SNP_ID	# Fluo.type	Chr.	Pos.	*E*‐value	Sequence
S1_78464789	4	1	1066677	8E−52	GTTTCCCAATGGTAACACTTTCTGCAAAGCCTGAAAGGCACTTGACTTGACATTGCCAAG[T/G]GCATTAGTTGCCACAGCCCCAATTCTAACTATAGCTGCAGCAGCAGCTAACGGTGAAGCT
S1_166177831	4	1	3002321	2E−40	CATCACAAGCGAAACAATGCAAGATCACTGCAGCGCTAAACAAGACGATGAAAACTGCTA[G/T]AACTGCCCACTCTTCCAAAGATAGACTGCAGCAAAACAAAAGCCGCTTGGATGATCACAC
S1_73817882	5	1	5214076	8E−52	TGATTCTTCTTCCTCTTCATCTGCAGACTTTTTGGATGATGCTACTTCTTCACTAAACAA[A/G]CAACCTCTTTATCAGATGTCTTCTATCAAGGCTGAACTTCCAATCAAGTTGTGTGATTTG
S1_308276216	4	1	22422993	2E−54	GAAGCTGATTGAGCTGCTTGATATCGATCTGCAGTGGAGGATGCATAAAGTTTCTGATGG[G/C]CAGCGTCGTCGTGTGCAAATTTGCATGGGACTTTTACAACCATACAAGGCAATGATTTTT
S3_3283493	4	2	6259005	3E−51	TCTCAAGTAACTATTATGGTAGTAACAGATGATGCAAATGTGAAGGCAAGATAAGAAATA[C/G]CATACCTCCCCATCTGCAGCACAAGTAACGAGGGTCCGATCATCTGTGTAAGGCATGAAT
S1_15620393	5	2	21404053	8E−52	GATCAACTGCAATGCCAATGGTTGGTGCAAGTTTCTTGGGAATACCTGCTGCCTGAAATG[T/G]AAAACCCGTACAATATGATACAAATAAGTGGAGTGCCTGTGCTGCAGCTGAGAATTGAGA
S1_194680124	3	2	25133441	8E−52	TTTTGACTGACAGCCTTTAGTGAACTGCAGGCTTACATGGAAAACCTCTTGACCTCGCTG[C/T]GAGGCTGGATATAGCAATTGATGTTGCTCATGCTATTACATACCTTCACATGTACACAGG
S1_149013038	4	2	29033873	1E−43	CTCTCAGGTATGAATGGATGGTGCCCAAATGATTTTGAAACGCCCACATGGGTTTGTTGA[A/T]TGTGTTATCTACGCAACCAAATTGTAATAAATGACTAAATGTGGTAAATCTTTTCCCTGC
S1_54908604	5	2	31946176	8E−52	AACTGACAAAATGGCAATGCAATGCCCTTTGTCACTGATCACAAAGAAGAGAAGACATAT[A/T]AGGGTATTTTTATGGAAAAACAAAGATGGTCCATCTTATTATTATTTTCTCCTGCAGGGG
S1_220358774	4	3	562364	8E−52	GGGATTGACAAAAGCACAATCATTTACATGCTGCAGATTCGGCAGATTTTGCTGCAGATG[G/T]TGCTCCACCATCATCATTGGCAAAGGGGTAGCCTGATTTCCATGGGACACTTGGAGAAAG
S1_294347489	4	3	3182257	5E−48	AGGAAGAGTATGTTCTCCATCAATTACATTCTCATTACGCAACTTCAATATATCCATCAG[A/G]AAAGGGTTATTCTGGTGAGCAATACAATACACATTTTCTGCAGCAGGAATAGAACATATG
S1_213496700	5	3	6608590	2E−53	TATATAAACATTCCATTTTGATGAGAATGAGAGACCATTGTTGCTAGCATCCCATTGACT[A/G]CCATATCTGCAGGGATCTGTATGGAAAAAGTGCATGCATGAAAGACAAATATAATAATAT
S1_123502462	5	3	12211409	2E−46	ATAGAGAAAAGACCTGCAGAAGCAGAAGCAACACGATCATCCTTGTTGACATCTCGCAAA[C/T]GAAGAGAAAGCTTTTTGGTGAAGTTTGAGTGAGAATTGTAGAAGTCCTCCATGGCCATGG
S2_13394502	4	3	12986600	1E−50	GTCTATTTAGCATGTCTTAGTTTCTTGGTGATGATGACTGCAGTTGAAGTCAAAATTTGA[G/T]GATCTCTCATCTGAACATCATCATGCTTGTGAAGAAATGAATAAATTGCAAGAAAAGCTG
S1_212477984	4	3	18808497	2E−53	TTGTAGTCGTAATCGAAATCGTATTCTTTGTGGAGTATTATTTTAGGTGGAAGATGTTGA[T/G]ATTTCTGAAAGAGTTCAGAGAGGATTAGAATCACCAGCCTACTGCAGTGGAAGATATGTA
S1_40517926	5	4	2442039	3E−50	TGGTTAATCGCAGATGGGGCTTGGAAAGACTCTGCAGGCGATGTCTTTGTTGAGCTATCT[G/A]AAGGTCAATGGCATCTCAACGGGGCCGTTCTGTGAGTCTTGGTTTATCTCCGATGAGCTT
S1_341690721	3	4	4172027	7E−46	TTGTGCATTCCTCCCTGCATCTCTTGGAACTGCAGCCTGCCCACTCCATCCTCCCATGCT[A/G]CTCTTGTGAACCATCTCAACCACTCTTCTTCTTTCTCTCTCTCTTTCTCTCTGTTGTCTC
S1_94822591	5	4	6518043	4E−37	TCTGATGATCGTGCTTCTCTCATCAAATGTTAGATGTTGTTCTAACTCTTCAAGCAATCA[G/A]GAACTTATTTGCTACTATGAGTTGTGACTTATTGTTGCTGGTCACTGGATACTGCAGGTC
S1_223059854	5	4	9590872	4E−49	GTTGCTGCCAGCAATCATGAGACCATTATGAGCTATGTTGATGGATGGTGAGGTTCGGGA[C/T]GTCTGTGGATAAGCTGTAACTGCAGGCAGGCTACCTACCATTGGAGAAAATTGGCCAGCT
S3_1940455	4	4	11602024	4E−49	ATGCCTGAATCTGGAGGACAAGCTACTGCAGTGTGTCAAGAAATAGACATACTTGAAGAG[A/C]ATTATGAATCAGAACAGTTCCAGGCCGGTAATGTTGAGTTCATGCTTTCTGTTCCTTTCT
S2_59089982	4	4	13022373	2E−47	CTCAATAATGGTGGACAAATGTGCCTTCTAATTCAGAAAAAAAATGTCTACTAATTACCT[C/G]TCCAGCTCTGTCATTTACTTGTTATAGGATGACATAATTGATGACTCTGCAGAGGATGAT
S1_29508975	5	4	13956140	8E−52	TAGTGAGCAGTTGAGATCATTAACGAGCATAGAAGAACTCACTGCAGAAGCCACAAAGCC[A/T]GAAGTCAATGGAGTTTCTATGGAACATAATGACGAGGAATTGGGAACACTATATTGCACT
S1_78099239	4	4	19009721	2E−33	CAGGTTTTCTTTTCAATTGCAAGAGACATCAAGCAAAGGCTTGCAGAAACCGATACCAAA[C/G]CTGAGGTAGTATGCTTATCATTTGTGATAAATTCAGTAAACCTGCAGGCAATTAGTAATG
S1_98182899	5	4	25059970	2E−47	TGTGCACAATGCTATGACCACACATATGGTTTCAAACAGACAAGGAACAGAATCAACAGA[T/C]ACACTACTTACAGTGACAAGCTCCGATATCAGTTGCGCAGACAACCCTGTTTTCTGCAGC
S1_13376874	3	4	25692581	2E−40	CTTGATATGTCTGTATTGGGTGTCATTTCTGCAGTATTATTTGGTCCGCAAAGGTAAATC[A/G]ATAGAAATTGGCGTCATTTAGCAATATCTAAACTGTATTTGAATTTGTAGTTCGAGCATT
S1_50863270	5	4	27382503	2E−54	GTGGCTCTCAAAGACTGAGGAAGTGCGTGAAGATAGGGCTCATTGGGGTACCAATATAAC[T/C]GGTGATATTTATGGTCAGGGTTGGATCAGTGAAATGTATGGATATTCATTTGGTGCTGCA
S1_30240813	5	5	4418552	4E−43	TCAGAGAGTGAGTCACATAAAAATAAGATTCATGTTGCATTGTGATGCCCCCTGATTCTT[A/C]TTACTTGCCCCCAAACGATAAGGACATCTTTTCTGAAAACTGCAGAGCCTAAGGAAATTA
S1_284257251	5	5	8809466	2E−41	AATTTTCTGATCTGAGTATTGGTCAACAAGAATCCAACATAAAACTCAAGTAAAATGCAG[T/C]AAATTACAATTGTTACATAATTGTTTCTCCACATAACTTGCTAATAATTATTTCTGCAGA
S1_172013713	2	5	15915339	5E−48	AACGCATGATACTCAATGTGTTGTTACTAATTGAATCTCAATTAATATGACCTGCAGTTG[C/T]TTGAATTTTCATGCTATGTTTGTAAGGCCTCTAGTGTTGCCAAAACCTCAGACATCTTCG
S2_46046547	5	5	18636385	5E−54	CACTGCAGGGGCTGCTTCCTTGATGATGAACCCAAAGAACTCTATTTCTCAAATAAAGCG[C/A]TTCATTGGAAAGAAATTCTCTGACCCGGAGCTTCAGTCTGACTTGCAGTTATTTCCTTTT
S1_161404508	4	5	19741536	5E−42	TGCTAATCAAAACTGATGCCTCTGCAGGGACCAAGTTGATCATTGAAAGAATCAGGGATT[A/C]TCACTTTCTATCACCTGTAGAGTACTGCATGTACATAAGTCACCATGAAAAGCACCGGGC
S1_86749745	5	5	20784276	1E−48	CCCTTCTGATATTTGCTTGGAGTTGAGATGTCTGTTGAACTTTAGCTGGAAATTTTACAG[T/C]CAACTCTATGAATTGTGTTTTCTGATTCATACGCACATTTGTGATTTTGTGCCTGCAGAG
S1_349430697	5	5	22888769	8E−52	TCCATCCTGGGAAGCACTGGCAATGGTTGATTTCGGTAGGCCAAGATTTGGAGCCCAAGC[G/A]ACATCTCTAACCCAATCGGAATGCATCTGCAGGGCAGGAAAGCAGTCCATTTTCCAGCTC
S3_43353096	5	5	24048351	8E−52	TGATGGGGGGAAATAACCCAAACTGGTGGAGTTCTATCAGCAACATGAAGCCAACTGCAG[G/C]AGATGAAACCTCTCTTCTTTATCCTTTTCCATCTTCTTCACCTCTCTTCCATCACTACTC
S1_51060666	3	5	26180154	1E−42	GAAGGAAGGCAGCAGCCTTTCAAACCTGCAGATGAAGTCGCCACGTCTTTTGAAAATTGC[A/T]AACCCAGCCAATTTTGCAGCTGCTAGTTTATAGGTAATGATAGATAGTTATCTAGGCTAT
S1_126396048	5	5	27405531	4E−49	CAACTCTGTCGTAGGAAAAGAAACTGACCCGTCCATGATCAACAAAACAGAATTTTAGAG[G/A]AATGCCAAGGCACTTCTTCTCAAGGTTTTCTAATTCTGTTCTTGAGGGTGGCTTGTCTCT
S1_189523417	3	5	30765836	4E−49	TTTAAAGCTTGTGACAGCGAGTTTGAAGACCTCTGCTGCAGGCATGCATCCAGCTTGCGC[A/G]GCAGGAACAGCTACACCGGCTGTTACTGCCGTGGCGCTTTCACTCAGTGGACTGAAAACA
S1_210690510	5	5	31577924	2E−53	ATCACCTGTCATTCTTAGCATACGCTGACAGCATCCAGCAATAAGCCATGATGCTGGGCA[A/T]GATTCCCAAGGACTGGATCGCTTGAAACTGCTCTTTACTCTCATCTACAAGCCCTGCAGA
S1_199164936	5	6	10295314	1E−37	ATTATTATTATTATTATTTCTTCTTCTGCAGTAACGGGTCACATTGCTTGGAAGAAGTTG[T/C]TGGAGCTTGAAACGCAAATAATGATACGAACACAGCCTCAGCAATGCACGATTACTCGCT
S1_282211588	5	6	17925427	2E−40	CACTTTGCACAATTATCTGCTGTAGAATGTTCTATTTGTTAAACCTGCAGATTAGGAAAT[C/T]CTAAATTCTATCTGCTGTTATGAAGTCCTGGTAGTATGTACAAGCAGGTTGATTATACAT
S1_116006917	4	6	21845952	8E−52	TTGTCAAGGAACGATCCCTTCACCTCCTCGGAGAAGAATCGCCCGAACACACGAGAGATG[G/C]ATATGGCCGGAACCTGCAGAGGAGAAAGCGAAGCCCTAACCCTGAGGTGCTTCAGCACAG
S1_45761963	5	6	27083504	1E−50	TTTTATCATGAACCGATCATCCTGAGACAGGTAGAAGAAGCTCCCACTCTTCCCAGGGGA[G/A]GATAATTCCCTCAAAGCATCACTTCCACAGATCGTCAACATATAATCTGCAGCATCAACC
S1_289563297	3	6	31210760	2E−53	AATGAACCATATCATAATCAACTAGATGTGAAAAAAGAATATTTGCACAACTGCAGGTGG[A/G]CAGGAAACCAAGGGGCTAAATAGACACACCTCATGACCTAGTTTCACACCCATCTCCTGT
S1_210284742	5	6	32022412	1E−50	AAAAGACCCAAGGAAATGACACAGCAGAACCATTGTCCCATTGGACATTTTCAACTACAT[T/G]CAAACTGCAGCATAAAAACCAAGATTTATATCACATATCCACACTAGTTCAATGAAACAA
S1_244041680	5	7	891269	4E−49	ATCAAAACATCGCTCTCTGCAGCCAAATCACAGACGTTAGAGAAATATTTATAGGCGAGT[G/A]ATGGCCTTGTTGTTCTAGAATGGTACAAGATTGTGCAGCCAAAGGCTTCGAGTCGTTTTG
S4_1831247	3	7	3180748	5E−48	GACAAACCAGAAATCTTTCCTTTCCATTAAGGAAGCAAATCCACCAAGGAGAACTGCAGT[T/A]GCCCAAATGAATCCGAGGGCTCCCAAACCACTGGCTGCCTTTTCAAGGATTGCCAAGCGA
S1_156520859	4	7	10367143	8E−52	TCTTTTACTGATATAAAGAGACTACCAGAATCCATTTGTATGTTGGTTAATCTGCAGACA[C/T]TGAAACTCTATTGTTGTTATAAACTTTCCGAGCTTCCCAAGAGCATAACATACATGAACA
S1_109907043	3	7	15658966	2E−53	AGGAGAAAAAATTCATGTGATGTCCTCCATATCTCAGCCTCGTCTCGGGTGGTCAAATGA[A/G]ACTGCAGCAAGTATTGGGACAATTGCAAGAATAGACATGGATGGCACTCTCAATGTGAGT
S1_365833705	3	7	17541018	1E−50	ACACATCTTCACCATTCAATCACTTTCATCCAACTGCAGCAACGTCTCAACAAGATCTCC[C/A]TGAGCTAGGTATCATCAATTTTCTACAAGCAATCTGCATTGGAAAGTGATCATGGACCGA
S1_215375978	5	7	17828247	4E−49	TGCTGTGCTCACGCCGATGGATACTGTGAAGCAGCGGCTGCAGCTTGAGAGTAGTCCGTA[C/T]AGAGGGGTGGGTGATTGTGTGAGGAGAGTGATGAGGGAAGAGGGGGTGCGTGCGTTTTAT
S1_5956960	5	8	1990861	5E−35	AGATAAGCACTTTGTATCTTGCTATTTTTGTTGCTCTTTATTATTGATGTGCAACAATGT[C/T]CCCAACAACCACACACACACACACACACACAATTTTGTATTTTTATGTTAGCTACTTCAT
S1_142832546	5	8	4073006	4E−49	AACTAACATGAATTTTGGCTCAATGATATAAGATTAACAACAAAAACGTTTTTGCTGCAG[G/A]GTTCTTGAACAAGTTTGATGAAATCACAAAATGGATATTGAAAGTTTGTAAGAATGTTAT
S1_102926938	3	8	5771893	3E−50	AATCTTTGATGACAAAGCTGCAGCTTCTTTTCATGCAAAACAATAAAAAGTATACCGGAT[C/T]TGATGTGATATGGGATGATCAGATCACTATACTGAAAATGAAACCTGTGCCAGCTTCTCT
S1_29231327	5	8	6476874	1E−48	TCCAGCAAATAGGTGGGGAACATCATACAACGGGCACGGAATGTTCATCGAAAATGCACC[C/T]GCTAACATCGTGGCCAAAGCTATGGAAAACATTGCAATAGAAAGTATAACCTGCAGCTGA
S3_54678463	5	8	7748403	4E−56	TCAAGAGCTTCAAGAAGAGGAAAGAAGGATAATAAGTGAAATATCAGAGTTAGAGTGTGG[G/A]AGACCTGCAGAAGAACAAAGAGTTTGTGTTACTGAAATGATGGATTGTGTTATTGATCCA
S1_208236889	2	8	9479207	4E−49	AGGAAGGAAAGAGAAAGAAGTTTCTGCTGCAGTCTCAGCCCCTTCTTCGAATTCTTCTTG[T/C]AGTTTATCAACAATCACTCAATCATACGGTGACAATGCCACTCTTCAAATAACTCAGCAT
S1_169356495	5	8	12133164	8E−52	ATACAGAAATTATCAGTGTAATATATTACAGAAGTAGAATGCTTCATCACCAGAATCTGA[T/A]TTTATATGAAAACACACTGACCTCTTGATGAAGAATTAGGCAAAACAGGGAGTCTGCAGA
S3_35309770	5	8	19352521	2E−47	CATTTCCAAATTTCAGAAAATAAATCGGTTTCCATAAGATTTGAGGTACAAATAGTTTCC[A/G]CAAAGGAGGTTTATCTGATACAACACTGCAGCTTGAATATGGTAAATAACTAGTCTCACA
S1_235419648	4	8	23125222	5E−48	GAACTTCAGAAATTGTTATACGCTGCAGATTGCCCAAAATGAAGCATTCATTAGACATAA[C/T]TGATCCCATAAATCAGCCCAGCTTCTTTTATGTTGTACATAAAAGTTCAATTAGCAAGAT
S2_30875426	3	8	27554786	1E−43	TTCATGTTTGGAAGATCTAATGTCAATTTAGATGTCATATGGTTTAGTTTTGTATTAGTA[C/G]TTTATGTTGTATTATTCCAATATAATCCAATATCATATCTGCAATTCTGCAGCAGGTCTT
S1_71285261	4	9	1039396	2E−53	AGAGAATGTCCGGATAAATCCTCAGAGAAGACTCCACCTTCGCAAGGTGCCCAGCTCGCA[C/A]GGCCATGAAGAACTCGAGCTCTGTGGGGTTACGGCTGCAGCTCAGGCCATGCCCCATCTT
S1_352413390	4	9	3168774	2E−53	CTTTCTTGGCAAACAGTTCTGCAGTAGATTTGAAGTCAGCTTCTTCTACAAGTCTACCAA[A/G]AGAGGATTCCAAGTCAGTGAATGGATATGATCAATTTGCATGACTGCTTGAAAAGTCGGG
S1_58454213	5	9	3570970	2E−53	TGTCCTGTGGCCTCATCGAGGAGCCATTTCTCTAGCATTGATAGAGGAGGGTTGTTATGG[C/T]TCTCAACTCTCTCCTTCCCTTCAGAATCACCATTGAAGATTGCTGATTCTGCAGATGATT
S2_9856110	5	9	5066064	1E−50	TGGAAAATTAGGTATCCCAGTTACCATGGAAATCGCTAGTGATCTGCTTGATAGGCAAGG[T/C]CCAATTTACAGAGAGGATACTGCCGTGTTCGTTAGCCAATCTGGAGAAACTGCAGATACC
S1_157448006	5	9	7773168	2E−46	TTTAACTTTTGAAAGGCTGCAGGGTATGAAATCACAGGCCCCGAGCCTGCTAATGTAGAT[C/T]ATGGTGAAGAAGCTGCATCTGAGGATGAAGAGGAATCTTATGATGGACATGATGCAGATG
S3_14699960	5	9	14771343	1E−43	AACTAAAAGCAAACCAAAAATAAATTCCTCTGCCGTTAAATAACCTGCAGAAAAAAATAG[C/A]GAAATGTGACAAGGAGAATATTTACATACCTTCGCCTCCATGACACTTCTTTCAGAGTTC
S2_58843160	5	9	17855377	5E−54	CAATTCATTTCAGGCATAATGTTATCAAGTAATGCATATTCTACCAGAAATGAACTTTAT[G/A]TGGAACATCATTCTTGACATTTGAAGAACTGCAGTTGATTACAAGTGAATTGCTTATAAC
S1_108505610	5	9	23138342	1E−48	GGAAAATATCCAAAAAGCAATAAAAGCTGCAATGGACGCAGCCAATGCCGCTGTTTCACA[A/G]TCGAAGGCATTCTGCCTAAGCCGCGTCGATGTGGGTCTAGACACCACTGCAGTTCGAGAA
S1_96409136	4	10	273696	9E−45	TGAAGTTTGATGATTCAATTTACCAATGATTTTCATGCACAGGAGTATATCTGAGAATAA[C/T]GAGCAAAATGATGCAGAGGTTACTTCAGCAAGAAATGCTGCAGAGAAGGATGTGACCAAG
S1_75907479	5	10	1295979	8E−52	AATGTCTTGACAGAAGCCATGAAAAAGCTCCACAAAATAAGTCCAAATTGAGATTGGAGA[G/A]CATACTCTCCACTGCAGCAAGTCTGTACCCTGTCTATGTGACTGCTGGAGGGGCTCTTGC
S3_17911820	3	10	2220748	8E−39	AGTAGTTTCTGAACTGGTACTTTGATCAATACCTGCAGAGTTAGTAGCAGTAGCAATAAG[C/T]GAGGAGACCTCAATATCTTGCACATGATCACTCACCGAAAATGGAACATTATCAGCATGA
S1_282032037	4	10	5002351	7E−40	GATTTACAGGACAATTACATTTCAGATTTCCATAATGATGGTAACTACAAGAATATTATT[C/A]TGTAAGCACCATGATACTTGTATCCATTACATGCATTGAATCAAAAGAACTGCAGTTTTA
S2_66969333	2	10	5976818	2E−52	TTTAACTGTATTGGCAGTGTCTGCAGACAGAGCTACGTCTAGCAAAGTGAGCAACTCATC[A/G]TCTGAAACAACTCCAATCTGTAAAACAGAGCAAGTCACAGAAAATTTATACCAAGATAAG
S2_53836832	3	10	10477461	5E−48	GAACACTTTCCTGGATTGAAAATTATTTCTGCTGCAGATCATCGTTTCTTTGGCACCACA[C/A]CCTTTTTCTAATAAAATATTCTTGAGCTCTTTCTCATCTTTGAGATGTGAAACATCTAAC
S1_21668183	4	10	16038685	2E−53	AGCTGCAGAAATTACATCAAGGATGGATCTCAGAGCTTCCAACCTGCATAACATCTCATC[G/A]ATTGCAATGTCATCAACTGGAAGGTGCCCTATGATCCCTGCCACGGTTGATCTCTCCTCT
S1_333790152	5	11	2622759	4E−56	TGCTTTGAAAGGCCAGCATGATCTTTTTTCTATTTTGTGTTCTTGCAATGAGTTCTGTCT[A/T]ACATTGCTGATTTTTGTTCTTGTGCAGTCTGCAGTAGATTTTGATGATAAAACCAGTTGG
S1_238131512	2	11	14447426	1E−43	AAATATCGAGATGAATTTCTGGGAACAAGCCTGCAGTTAGTTTGAGGATGTGTTTGGAAT[A/T]AGTGATCAAATGGCATTTGAGAAGCATAGTATGTAATTTTTCCGTAAAAAATGATCAAGA
S1_38918393	3	12	17162245	2E−46	AATCCCATCAAATTTGTCTTTCAAATTTCTGAATTTTTTCCCCTATCCCAAAAAATTCAG[C/G]CCATCAAGAAAATATGAGGCAAAGCATAAAACTGCAGCATAATCTCTATTAGATCTCATC
S1_102041015	4	12	24327364	3E−45	GCAGTATACAACTTCATTACCATGTTAGACCAGCAAATCTCAGTCTGATTTCTTCCTAGC[T/C]AACACACACACACACACACACAAAAGAAAACTTCAATCTCTGTTTGTTTTCTGAGTGCAT
S1_65460849	4	13	1824700	1E−42	GCATCTGCTAGTTCAAAATAAAAGGCAAGCATGGTATCACTAATACTGCAGAAAAATGAT[A/G]GTGCATAAATATCTAATGGGAAATGATGCAGCGAAGATCAATAACTTAGAATGAAATTCA
S2_23695000	5	13	8252985	8E−52	TACATCTGGGGGACTGCAGAGCTTTGAGCATCCACTGAACCTGGTGAAACCAGTGCCCAG[G/A]GTTGACAGCAAAGGGCAAGTATGTGGTGCCAATTTCAAAGTTGATGCCCAAGCTAAGAAG
S1_279910017	5	13	27313051	2E−53	GGCAGCTTCCAGGGCTCGGGAGCTACCAAGATGGAGTTTGCTATCCACGGATTTGTTCCA[C/T]GAATCTGTAGCTCCATCGTATACCCTGAGCCTGCAGCCGTCTCTGCAGTCCAGAGCATAA
S1_297529258	3	14	3933202	7E−46	CTTATCTATGGCCAGGTTCATCAACATATAAGGCTTCAATGGTCTCAAATTTCATCGGTG[A/C]TGCTTCTGCTGTGTGTACTTTTACTTGTCACTTACGCCAAAGTAACTGCAGCCTGCAGGT
S1_252699764	5	14	12391182	4E−49	ACTACTGAATAAATGGAATCAACTTTATTTGCTGCAGTTGGACTAGCCTAAGAGGAACTA[A/T]GTGGCTTTGGAAGAGTGTTGATACTTGGGATTTTATATCAATGTGTGAAAATCAGTGACT
S1_64347285	5	14	12868762	1E−50	GGATCAAAGTTCTCAGAGATTATTGATTTTGAGAAGTCAAGATATGCATCAAATCCGTGG[T/G]TGATCTCTACTGCAGCATTTACCATCCCTTTCATGTCAATTGACCAGAGAGGTGTCAGAT
S1_108652759	4	14	13736821	3E−45	GAGGTAATCTGTGACTTGTCCATATTACTGCAGAACAGCAATTTATTGCTGATCTTGGAC[T/C]ACTGATATCCAGCTTTCCCCAGATTATGTCATATTGCACCCAGCAAACCAGTACAATTTA
S2_68074878	5	15	320619	6E−47	CACCACCATCACATCCGCACCATTGTTGTCCACCTTTGAACCCAGTGTGCCTGAGAAGGA[C/T]ACCTGCAACATATCCGGTGATGACTTCTGGAAAGTTGCCTGCAGGGCAGATTAATAAAAT
S2_15031349	4	15	2754888	3E−50	TAAGAGAAAAATAGCTTACATTATCTGTGTCGACCTCTGATATAATCTCTTTGATTGTAG[T/C]GGCATCGCCCATTCCGTATTTCTCCATGGCTGATTCCAACTCATCTCTTGTGATATAGCT
S1_361168697	5	15	3199245	2E−54	ATTACTAAGATGCAATTTATAAACATATCTGCAGTTCATTGCTCTTGAAATCTCTGCACA[A/G]GCAGAAGAAGTTGAGATTTCCGTAAAAAAGCCTGAAAATGGTGGTAGTGCTTCAGAAGAG
S1_42812024	5	15	3456280	8E−52	GAACATTTTATAGTACCTCAAGGGGAGTGCCTTTACTGTCAAGAGGAAGGGGAACACCAA[C/T]TCCGCATCCTCTCGGAAATCTGAAGCAGCTAGGCCTGTCATCAATGGCTGCTGCAGTAGC
S1_31917523	5	15	3839679	2E−54	TTTACATCTATTGAACTCTCTGCAGGTTGAATCTGAAATATTTTGCTTGCATGGTGGTTT[G/A]TCCCCTTCTATTGAGACCCTTGATAACATACGCAATTTTGATCGTGTTCAAGAGGTTCCT
S1_16064479	5	15	5173749	1E−50	GCTCAGAAGAGCTCCATATGTAAGTTGGTTCTGGACTACCTCCGGGAGACTATTGAAGTA[A/T]CTCTCTGCAGCATCTACCCCCTTGACTTTGGATATAAGATCTATTCGTATTGCATGGGTC
S1_116148629	4	15	6642827	8E−52	GCAGGAATACAAGAGTATTGCCAGAATGAGGCTGGTACATATTAGCCAATCTCCGAATCC[A/G]AGTCATTTTACAGTTCTTGTACGTTCAATTCCAAAATCACCTGAGGAATCATACAGTGAT
S1_359692995	4	15	8357684	4E−49	GTGGCTGTCTAATTTGCAGTACTGCAGAAGTAAATATGAAAAAACATGAAATGATAATCA[C/T]AATTCATTGACTAACCTGTGCAGATCTAGAATAGTAGATGTAGGAGCGATTCACTTCATC
S1_290181714	4	15	9566725	2E−54	TCCTCGTATGGGGCCCAAGAGGGAACTCAAGTTTGCTCTGGAATCCTTTTGGGATGGGAA[G/A]AGCAGTGCTGAAGATCTGCAGAAGGTTGCTGCAGATCTCAGGTCTTCCATTTGGAAGCAA
S1_282323032	4	16	4930222	4E−49	ACTTGGGATTGTGATGATCAGTGTGTCACTATTTTGTGCTTATGTCCTTTGTCAAAGAAA[C/T]CGTAATAGTTCTGATTCAAAAACAAATCAAACTGCAGGTATTGCTTTCATTTGGATTTGA
S1_262914420	4	16	8184285	4E−49	AGCAGCACTGTCAGCAAGAAAACTATAAACCTGCAGACGAGAATATAACTAACATCACCA[T/A]GAGACTTCAAAACAACAATTTTAGTCAACAAGGTTCAAGAAAACAGAACAAGACCTTGCA
S2_63978772	5	16	14079687	2E−53	AAATACTGGTCATAAATATTAGATCACACATATGTGCCAGTGTGGTCAACAGATAATGCG[C/T]TGCTGCAGTAAAAGAAAGAACTTCAGCAGTGTGGCTAACAGACCTATTGTAACACTAGCA
S2_42975314	5	16	19099744	5E−54	CTGGGCCACGAGGGGAGGGCTGGTGAAAACTGACTGGAATAAAGCTCCCTTCACAGCCTC[G/A]TACCGAAACTTCACTGCTGATGCTTGTGTTTGGTCATCTGGCGCCTCAAGCTGCAGATCA
S1_349651036	4	16	22229815	5E−54	GCTTTGGAAGATAAAATTCAATCGAAAGTCAGAAGAAGCAAGCAAATTACACAATTCGTC[T/A]GCTGCAGTTCGTCCTCTAGTTCATCCACAGGCCCTTGGATATGACAACTCCCTTCCAGGA
S1_131821504	4	17	11148474	6E−41	ATCACTAGTACAATGCAACATGACAAAACCTGAAGTGCTATACAAGTGACTGCTTATTTC[A/G]AACAGGACAAATCTGAAGCATAGCTTGTAATACTTCTGCAGAAAAATAATGGCAAAGTTT
S1_305511589	5	17	13065352	5E−48	TGATGTGTAGATATGAGTGGAACTGATTTTTATAACTTTATTACAAAGCATTATTTTTTA[T/G]CCATCTAATGTTCTGCAGATTAGGTGGATGCATTTTTTTTAATAATTTTTTTAGCAATTT
S1_162377692	4	17	14256863	2E−53	ACAGAAGAGAAAGCTTGATGAGATGTATGATCAGTTGAGAAATGAGTATGAGTCAGTGAA[G/A]CGATCAGCTATACAACCTGCAGGCAACTTCTTCCAAAGAGCTGACCCAGACTTGTTTTCA
S1_7095019	5	17	18481610	3E−44	CACGGGTGAGCCATCAGTAGTACCTGATGCCAATGTTGAAACCATCGAATTCCCTTTCAC[C/T]GACTGCAGGTATACGACCTATAGAAAGGTTGCGGAATTAGTGACTACTTTTTTTTTTTGT
S1_164752250	5	17	19197258	1E−50	CGTTCAACAGCAAACTGCAGAGAACATCAATCAGATGATCGGAGCGAGCTCATCAGCAGA[T/G]AAAGCAATTGATGATTGTGTTTCAGGTTTTGATCCAAGCATCAGTGAGGACCTGTTCCAG
S1_104430414	4	18	7027601	6E−47	GGTACACACTGCAGACCTGGAGTCTTTGTTCCCTCTTATAACATGAGAGCTTGTTCTTCT[T/G]GTCATATACTTGCTGAAGCTTTTGCAACTCTTTTTACATCTGAAGCCAATTTCTTTACCC
S2_23048737	4	18	14902976	8E−52	TGATTATGCTCGCCATTTCTTGTCCTGCTGCAGTAGAATGCTTGGCCTGTCTTATGAATC[C/A]AAGAGAGGCTACATTGGTTTGGAGTACTATGGCAGGACAGTGAGCATCAAGATTTTGCCT
S1_259660238	3	18	18609390	8E−52	TTGTAGGCATCAATCTCCAAGAAATCATTATCTATTTGATGGTGTAAGTTCTTGTTGAGG[C/T]TGATAACTGCTGCAGCCTTCTCTTAGTTGAAGCATAACCTTTTTTTCTATTTTCCCTTTT
S1_170367846	5	18	21499070	8E−52	CCATGTCTCGGCCTGCAAACATTGGATACCAAGAATTATAAGAAATGGAAGTGAACGGAT[G/A]CGATGGTGAAAACATTTCAGTACCTGAAACTCTGCAGTAAGCACTCTTCTGAATGAGTTC
S1_31156518	5	19	1787283	2E−53	AGGACGTCCACACTTGATAGTGTTTCCATTTGCATCATGTTCATTCCACATGAATGCCTC[T/C]GGAGACATGTAGTTCAGTGTGCCAACCTGCAGTTTAGTAACATGAAATGACATACTACAA
S4_1766101	5	19	2912318	2E−41	TTCAGATGGATCAGTCGTAGATTGAGCTTTGAATCTCTGAACAAGGAGCATGACGCATAG[G/T]AAGCGGAAGCAGGAACAGGAGAAGGGAAACATGATCTGCAGCTGCAGCACCATCATAAGA
S1_130386685	5	19	3858772	2E−54	AGAGTTGATTATAATTTTATGATCACATAATAATTGAACAAAAACTGCAGAAACAATACC[A/G]GCTAAGTCATTCAGCACCAAATTATTTAGAGGTAAGTTTTGATTACCTTCAACTTTCAAG
S1_66878388	4	19	4662624	6E−47	AATTGTGACATCAGCAGCAATAAAATTTTTGAAAAACCACCGCCCAAAAAATTTATCAAA[A/G]AAAATTATCACTGTCTTGTGTCAAACATCATCACCAACTCCAAACCCATACTGCAGAAAA
S1_48170206	4	19	8029141	3E−50	ATTATACAAACTGACAAACGTATTCTATATCAAAATTGAATTACAGGAGAGATTGGTAGG[A/T]CGTAAAGATCCAAACATAGAAATCAAGATGTTAGAAGGATATGATAGAAACAACTGCAGA
S1_85054393	4	19	9504285	1E−50	CACATCTGTTTTCTTCCTTGAGATTTCTACAATACAACCTGAGAAGTTCTGCCAAAAATT[G/A]TCTCTTTGCGGGGTAAAATATTTTTCTCTCCATAGGAGTGACAACAATATCTGCAGGCTT
S1_16428287	5	20	725184	8E−52	CAACGTACTGCAGACCCTGTCAAAATGCCATGAATAGGTTATTATAGTTATGAACATCTC[G/A]TCAAACAAAGATTTGCAACCAATGGTATGAGCATTCAGATTTCAAGACTGTCCAAAAAAC
S1_43488339	5	20	2904117	1E−50	GACAAAGTCATCAAGTAATTGCCTTCTCATGACAGATAAATCTGACTGCAGGGATGTGCC[G/A]AGCTTATAAGGCCAATTAGACAGCATAAAACAAATAGAAATGCTGGTCATTGAGACAATG
S1_114295977	4	20	6774493	1E−48	GCTTGGCATGTTTATGATGAGTATTCCTTTTGATAGCAGTAAAAATAGTACTTATTGGCT[A/G]ACTTGAAAGATCTAGAAGATTGGTTGGACTGCAGTTATATTTTTCTCTAGCTCAGCTAGG
S1_30976509	3	20	8500327	2E−54	ATTGTTTGGCTCCATCAATATAGGAAGATATCTAAGGAGTTACTGACTTGTAATATGGAA[C/T]CATAACTGACAATATAGCTTCAACATGATGTACTTACTTCGTGAATGCCTGCAGAGGAGG
S5_17384653	5	20	8742611	1E−36	TTAACCATTAAAATTTATTTACAATGTAAAATTTCCACAGAGAGTAGGTTCTGTAATCTC[C/A]GATTTTCTTCGACAAAATCAGCGGATTCATTCTTTATTCTGTTTATTGTGCTCTGCAGCT
S1_160073245	4	20	9514825	1E−42	TTCCGCCTGATGCTTTAACATATAAAAAGCTGCAGATGCAGAAACTAAATATCTGACATG[A/G]TCAATAGTTAGATTATGACTTTCAGCTTGTAGATTACCAATTACCAGACCACCATAAAGC
S2_46633476	3	20	9808073	1E−50	AGATCATGTCGTGAAAATACCTTCAGATCCTTCTCCTCCTCCCCCGTTTCCCTCGCGTCC[T/A]CCAAATTCGCCTTTCCTTCCTCCACCACCTCCGCCTCCGCCTATGATGAGCAGCAGTGGA
S1_231563096	5	20	9944570	4E−49	GTTGAGTTTCAGAGTTTCTGCAGCTCCCTTTGCAGTAGTTAGGCAGTAACCCATCACCTG[A/G]AAAGCATATCGGTTTCGATACTGTATGAACTTCCACAAGAACAGCCCTGCCATTGCTCCA
S1_60794376	5	20	10523563	7E−46	TTCAATACTTGTTGTCATTATAACATCAGAGATTGCAATTCCTTCATGATGCCACTGCAG[G/C]ATCCCCATATGGACAGCTGGTTTCAGTTGTCATTATCCTATTGCACTCAAATCTTGAAAT
S1_199666449	5	21	240474	4E−49	AGTTCCACTCATAAAACCAGTTCTCTACCCAAGCGGTAGCACGAATTCCTCAACAAAATT[A/G]TAGAATTTCCACTTTGTTCTCCGGAAGCATTGCCATTGGTGGTGCTGCAGTTACTCCATA
S1_257000706	2	21	928527	2E−54	TACAAAATGAGATGGTTCACAAGCTGAAGAACAAACCTCATCTGCAGTAAAAAGAAGAAC[A/G]TCACTGTGCCTCAAAAAAAGTTCAAGAGCTCTCTGTTGTTGAGGCAGCCCAAGCAAAAGA
S1_171090323	5	21	2104297	1E−50	CGAGGGAAGGAGAATGTGGAGCTACTGATTGCAGATATACTTATTGGTTCAGCAGACTAC[G/A]TCCATCATGACCCCTATAATACATTGTTCTGCAGGCAATATCAGTGTTCTTCCTAGGACC
S1_25663258	3	21	2772381	8E−33	TGTGAAAATTTGGCTGTTTTGCTGCAGTTTCATCATATTGCAAACTAATATTATAAACTC[A/T]AAAAGTTTTGTCCACTATAGAACTAGTGTCAGCATCAATTTCCAATATTAGCCAGGCTAA
S1_371159620	4	21	3932249	1E−50	GTCTGCAGGGGAGGTCAAACTCGAGCAGGTTTCGCATCAATGAGTGTAGCACCTTTATCA[C/T]CAGCATTTATTCCGGCTGCTGCCGCCACCACTCATGTTGGACTGCATCCAACAATCCTGA
